# Effect of Plastic Deformation on Thermal Properties in Twinning-Induced Plasticity Steel

**DOI:** 10.3390/ma17215263

**Published:** 2024-10-29

**Authors:** Joong-Ki Hwang

**Affiliations:** School of Mechatronics Engineering, Korea University of Technology & Education, Cheonan 31253, Republic of Korea; jkhwang@koreatech.ac.kr; Tel.: +82-41-560-1642

**Keywords:** thermal conductivity, twinning-induced plasticity steel, wire drawing, plastic deformation, grain elongation

## Abstract

The effect of plastic deformation induced by wire drawing on thermal properties in twinning-induced plasticity (TWIP) steel has been investigated. The investigation on the relationship between thermal conductivity (*k*) and the microstructure in the drawn TWIP steel wire was systematically performed to accurately understand the behavior of the *k* of a metal during wire drawing. The yield and tensile strengths linearly increased with drawing strain owing to the deformation twins and dislocations that were generated during wire drawing. However, the total elongation sharply decreased with drawing strain. The linear thermal expansion coefficient of the TWIP steel exhibited a similar value regardless of drawing strain. The density decreased linearly with temperature, and it was independent of the drawing strain. *k* increased initially and then decreased after reaching its maximum value with increasing drawing strains. At a nominal drawing strain of 0.26, *k* increased compared with the state of hot rolling because the increase in *k* due to grain elongation was greater than the decrease in *k* due to dislocations generated during wire drawing. However, as the amount of drawing step increased further, the influence of dislocations on *k* increased more than that of grain elongation, causing *k* to decrease.

## 1. Introduction

The weight reduction of automobiles has been continuously demanded to improve the energy efficiency of cars and prevent environmental pollution. Recently, the demand for the weight reduction of cars has become stronger owing to the maturity of electric vehicles. In this situation, researchers in steel industries are constantly studying to develop stronger and lighter steels. In particular, researchers’ efforts are accelerating to avoid losing market share to other materials in car industries. This trend is also apparent in steel long products industries such as wire, rod, and bar industries, which produce springs, tie rods, bearings, bolts, tire cords, etc. for automobiles. Practically, the most effective way to increase the strength of steel is to control its chemical composition. Accordingly, research and development on steels has been directed toward increasing the chemical composition of steels to effectively use their strengthening mechanisms such as solid solution hardening, precipitation strengthening, and strengthening with heat treatments. However, as the chemical composition of steels increases, the thermophysical properties of steels tend to deteriorate. This may cause some issues during the manufacturing process and/or under service. For instance, Hwang et al. [1,2] have suggested twinning-induced plasticity (TWIP) steels as good candidates to replace existing steels for wire, rod, and bar products because TWIP steels satisfy the products’ property requirements such as high strength, ductility, and resistance to hydrogen-delayed fracture [3,4,5,6]. The excellent mechanical properties of TWIP steels are attributed to deformation twins and/or dynamic strain aging (DSA) occurring during plastic deformation [7,8,9]. In TWIP steels, 10–30% manganese is used to stabilize the austenite phase. This high alloy tends to deteriorate the thermophysical properties of steels [10,11], which can worsen the formability of TWIP steels, in particular the wire drawability for wire and rod products. The wire drawing process is almost an essential process for producing wire and rod products such as springs, bolts, tie rods, tire cords, and bearings [12]. During the process, the temperature of the wire increases because the drawing speed is significantly high for productivity and wire drawing is performed through direct frictional contact between the wire and die [13,14].

The quality and productivity of products are closely related to the heat generated in the material during wire drawing. For example, temperature rises and thermal gradients in the materials can worsen the formability of the wire, and finally may cause wire breakages during the drawing process in plain carbon steels due to strain aging of the drawn wire [15]. The heat and thermal gradient in a material generated during wire drawing is highly correlated, not only with process conditions, but also with the thermal properties of the material [16]. For example, when the thermal conductivity (*k*) of a wire is low, the drawing speed should be reduced and more attention should be paid to cooling the wire during the drawing process. This is because the surface temperature of the wire increases with decreasing *k* and the thermal gradient in the wire increases. This means that process conditions for wire drawing should be modified according to the *k* of a wire and the productivity of the wire drawing process is dependent on the *k* of a wire. Therefore, to effectively design the wire drawing process, we need to accurately understand the thermal properties of metals, especially *k*.

In addition, since most wire, rod, and bar products are used after the wire drawing process or undergo additional processes after the wire drawing process, it is of great importance to understand the thermal properties of the drawn steel wire [1]. Consequently, it is necessary to investigate the thermal properties of the drawn TWIP steel wire to improve the drawability and productivity of wire products. To the best of the author’s knowledge, no studies have been conducted on the thermal properties of drawn TWIP steel, although there is considerable research on the drawing process conditions and mechanical properties such as drawing speed, drawing direction, die angle, drawing force, strength, ductility of materials [13,15,17,18,19,20,21,22,23,24] and electrical conductivity in Cu and Al alloys [25,26]. Meanwhile, it is known that the thermophysical properties of metals such as thermal diffusivity (*α*), specific heat capacity (*c*_p_), linear thermal expansion coefficient (*β*), density (*ρ*), and *k* are strongly dependent on the temperature [11,27,28,29,30].

Consequently, this study investigated the thermophysical properties of TWIP steel wire, especially *k*, during wire drawing. The thermophysical properties of the TWIP steel such as *α*, *c*_p_, and *β* were measured with temperature and drawing strain. Subsequently, *ρ* and *k* were calculated based on the measured thermophysical properties. The investigation on the relationship between *k* and the microstructures in the drawn TWIP steel was systematically performed to accurately understand the behavior of the *k* of a metal during wire drawing.

## 2. Experimental Procedures

### 2.1. Material Preparation

An ingot of 50 kg was cast using induction melting for the specimen preparation of Fe-Mn-C-Al TWIP steel. The alloy component of the ingot is listed in Table 1. Although there is an excellent combination of strength and elongation in TWIP steels, great efforts have been put into further improving the formability and resistance to delayed fractures in TWIP steels. Researchers showed that the formability and resistance to delayed fractures in TWIP steels increased with Al addition in Fe-Mn-C TWIP steels [31,32,33,34,35]. The addition of Al contents in TWIP steel improved the yield strength (YS) through the strengthening mechanism of solid solution, formability by suppressing the DSA, and resistance to delayed fractures. Meanwhile, C stabilizes austenite and inhibits martensite transformation by increasing the stacking fault energy (SFE) in TWIP steels and the addition of C contents in TWIP steel dramatically increases the YS through solid solution strengthening [36,37]. Therefore, Fe-17Mn-0.7C-1.5Al TWIP steel was selected as the test material in this study as listed in Table 1.

To suppress manganese segregation in the ingot, the cast specimen was heated to 1200 °C and homogenized for 12 h using a furnace. Then it was directly hot-rolled into the plate with the thickness of 20 mm. The rolling temperatures ranged from 950 °C to 1200 °C. Subsequently, the specimen was cooled in an atmospheric environment at a temperature of 21 °C. For a wire drawing test, several 15 mm-diameter round rods were machined from the hot-rolled plate using a lathe. The SFE of the present TWIP steel, calculated using the thermodynamic model proposed by Saeed-Akbari et al. [38] and Dumay et al. [39], was approximately 31.2 mJ/m^2^. Accordingly, this TWIP steel is expected to be deformed by a dislocation glide and form a deformation twin during the plastic deformation at room temperature (RT). In contrast, martensitic transformation is strongly suppressed during deformation due to the relatively high SFE [40,41,42].

### 2.2. Wire Drawing Test

The TWIP steel wire was drawn at a velocity of 8.3 mm/s using a draw bench machine with a single pass at RT. To clean the surface of the specimens, pickling was conducted with 12.5% HCl before the test. A MoS_2_ spray-type lubricant was utilized to decrease the frictional stress and improve wire quality during the drawing process. The specific die designs used in this test are summarized in Table 2. The reduction in area (*R_A_*) is calculated as follows, and the *R_A_* per pass was designed to be approximately 10%.

(1) $$R_{A}=\frac{A_{i}-A_{f}}{A_{i}}\times 100\;(\%)$$

Here *A*_i_ and *A*_f_ are the initial and final cross-sectional areas of the wire, respectively. The nominal drawing strain (*ε*_N_) of the specimen is obtained as follows and is summarized in Table 2.

(2) $$\varepsilon_{N}=ln\frac{A_{i}}{A_{f}}$$

### 2.3. Measurement of Microstructure and Mechanical Properties

**Figure 1:**
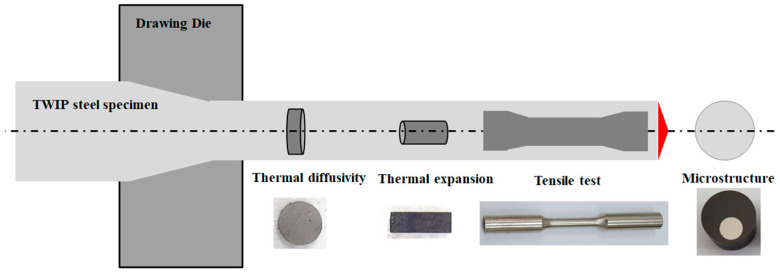
Schematic description of specimen preparation in drawn wire and a photograph of specimens for evaluation of thermal properties, tensile properties, and microstructure.

The samples were sectioned perpendicular to the radial direction of the hot-rolled and drawn wires using a cutting machine to observe their microstructures as shown in Figure 1. Microstructures were evaluated using electron backscatter diffraction (EBSD). Based on the EBSD techniques, the grain size and orientation, phase, density of deformation twin in the TWIP steel and the geometrically necessary dislocation (GND), etc. can be easily analyzed. In this study, we investigated the comprehensive relationship between *k* and the microstructure in the TWIP steel wire with applied strain induced by wire drawing. Therefore, EBSD measurements are the most suitable method for microstructural evolution in this study compared with a scanning electron microscope or an optical microscope. For the specimen preparation, the samples were progressively ground using SiC papers with water up to 2000 grit. Grounded samples were polished using diamond compound pastes from 6 to 1 μm; subsequently, a colloidal silica suspension was utilized for approximately 1.2 ks. A field-emission scanning electron microscopy with a TexSEM Laboratories (TSL) EBSD system was utilized to gather the EBSD data at an acceleration voltage of 20 kV. The measured area of the EBSD data was 180 μm × 180 μm with a 0.1 μm step size. The sample tilt angle was approximately 70°. The obtained EBSD data were evaluated using orientation imaging microscopy software.

For the tensile test, cylindrical-type specimens with a length of 25 mm and diameter of 5 mm were machined using a lathe along the hot rolling and drawing directions as shown in Figure 1. Tensile specimens were pulled at an initial strain rate of 10^−3^ s^−1^ with an Instron machine at RT. Ductility of the specimen was measured using a mechanical extensometer during the straining.

### 2.4. Measurement of Thermophysical Properties

The thermophysical properties of metals such as *ρ*, *c*_p_, *α*, *k*, and *β* are dependent on the temperature and applied strain. Therefore, the *α*, *c*_p_, and *β* of the TWIP steel were measured with temperatures and *ε*_N_. Then, *ρ* and *k* were calculated based on the measured thermophysical properties. The α was measured with laser flash analysis (LFA), Netzsch LFA 467 HT, Germany. The LFA specimen with a disk shape in a diameter of 10.0 mm and thickness of 2.5 mm was utilized as shown in Figure 1 [43]. During the test, argon gas was used to suppress surface oxidation of the specimen. Measurements were conducted at the interval of 100 °C from RT to 1000 °C.

To calculate *ρ* using temperatures based on *ρ*_0_, the thermal expansion ratio (Δ*L*/*L*_0_) was measured with thermomechanical analysis (TMA), TA Instruments TMA Q400, USA [44]. *ρ*_0_ indicates *ρ* at RT, and it was measured using the Archimedes’ principle at RT. *L* indicates the length of the TMA specimen. The subscript o indicates the values at RT. The specimen with a cylindrical shape in a diameter of 5.0 mm and thickness of 12.0 mm was utilized as shown in Figure 1. Nitrogen gas was utilized to suppress air intrusion during the test at a heating rate of 5 °C/min. Measurements were conducted from RT to 950 °C, and *β* was mathematically calculated using the following simple equation [45].

(3) $$\beta (T)=\frac{∆L}{L_{0}}\frac{1}{∆T}$$

To calculate *ρ*, the volume of a specimen (*V*) can be mathematically calculated as follows.

(4) $$V=L^{3}={\left(L_{0}+∆L\right)}^{3}={L_{0}}^{3}{\left(1+\frac{∆L}{L_{0}}\right)}^{3}$$

At a small Δ*L*/*L*_0_, Equation (4) can be approximated as follows using the Taylor series expansion.

(5) $$V=V_{0}(1+3\frac{∆L}{L_{0}})$$

Accordingly, *ρ* can be obtained based on Equations (3) and (5), and it can be expressed using the measured *ρ*_0_ and *β* as follows.

(6) $$\rho =\frac{m}{V}=\frac{m}{V_{0}(1+3\frac{∆L}{L_{0}})\;}=\rho_{0}\frac{1}{1+3\beta ∆T}$$

The *c*_p_ was measured with simultaneous thermal analysis (STA), Netzsch STA449 F5 Jupiter, Germany [46] from RT to 400 °C. To suppress surface oxidation of the STA specimen, argon gas was used during the test. Finally, *k* was obtained using the *c*_p_, *α*, and *ρ* as follows, according to the definition of *k* [47].

(7) $${k\left(T\right)=\alpha (T)\rho (T)c}_{p}(T)$$

As explained above, *α* and *c*_p_ were measured using LFA and STA, respectively, and *ρ* was calculated based on Equation (7).

## 3. Results

### 3.1. Microstructure

**Figure 2:**
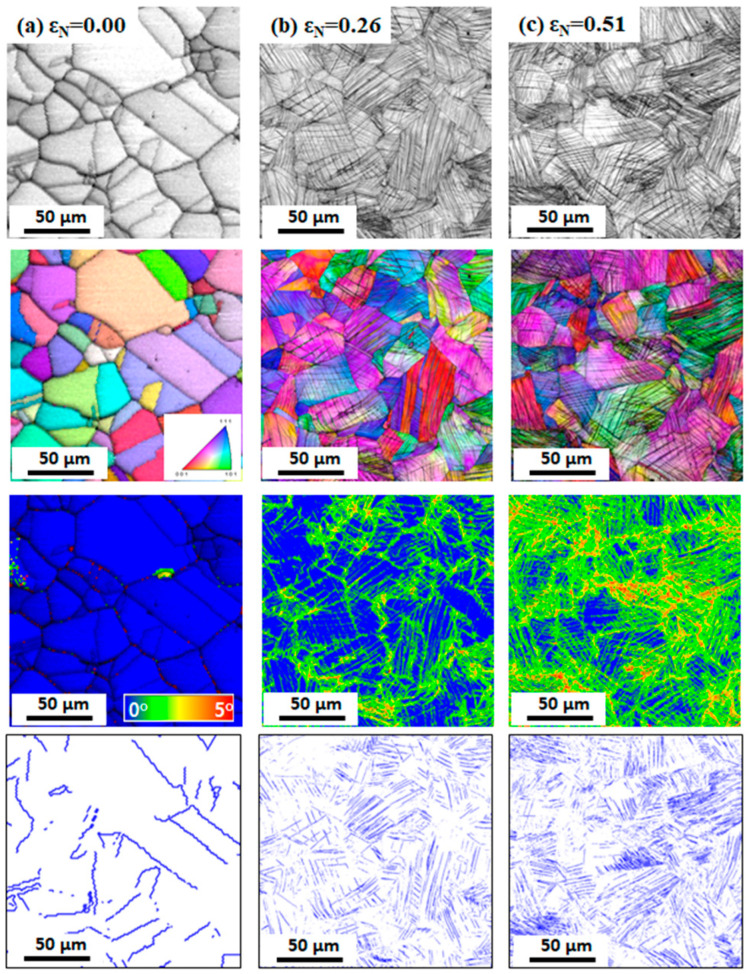
EBSD image quality, inverse pole figure, kernel average misorientation, and twin boundary maps of TWIP steel at nominal drawing strains of (**a**) 0.00, (**b**) 0.26, and (**c**) 0.51.

Figure 2 shows the comparison of microstructures of the hot-rolled and drawn TWIP steel specimens based on the EBSD analysis. The EBSD image quality (IQ), inverse pole figure (IPF), kernel average misorientation (KAM) and twin boundaries maps are compared with *ε*_N_. In twin boundaries maps, twin boundaries are defined as the misorientation angles of 58° < θ < 62°, and they are presented using blue lines. In the hot-rolled specimen (*ε*_N_ = 0.00), fully recrystallized grains with no significant textures and no GNDs were observed. It is known that the KAM value highly depends on the GND density [48,49]. Annealing twins were observed, but no deformation twins were seen. The average grain size was approximately 37 μm. At an *ε*_N_ of 0.26, most of the grains exhibited deformation twins, and the GND density increased compared with the hot-rolled specimen. The amount of deformation twins and GNDs increased at an *ε*_N_ of 0.51. In particular, deformation twins with multi-variants were highly observed at this drawing strain. It is generally accepted that deformation twins with more variants effectively hinder the dislocation glide due to the strong dynamic Hall–Petch effect, resulting in the outstanding work hardening rate of TWIP steels [50,51].

**Figure 3:**
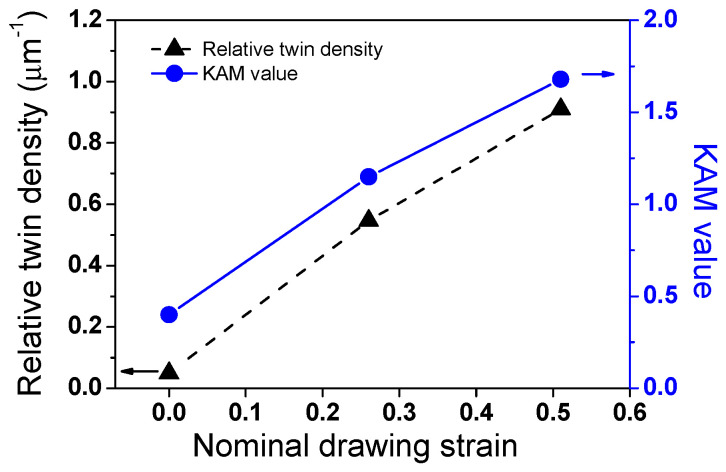
Comparison of relative twin density and kernel average misorientation values with nominal drawing strain.

Figure 3 shows the comparison of relative twin density (*D_twin_*) and the KAM values with *ε*_N_ for a more quantitative analysis of the microstructures. To calculate *D_twin_*, the total length of twins (*l*_twin_) was obtained based on the EBSD technique, and it was divided by the measured area (*A*_m_). Accordingly, *D_twin_* can be obtained using the following equation.

(8) $$D_{twin}=\frac{\sum l_{twin}}{A_{m}}\;(\mu m^{-1})$$

It is clearly shown that the D_twin_ and GND density increased with increasing *ε*_N_. The KAM and *D_twin_* values showed a similar trend with *ε*_N_, meaning that the GND density is related to the twinning behaviors. Unfortunately, we cannot determine the specific pattern of growth in the KAM and *D_twin_* with *ε*_N_ because only three points of *ε*_N_ were measured in this study.

### 3.2. Mechanical Properties

**Figure 4:**
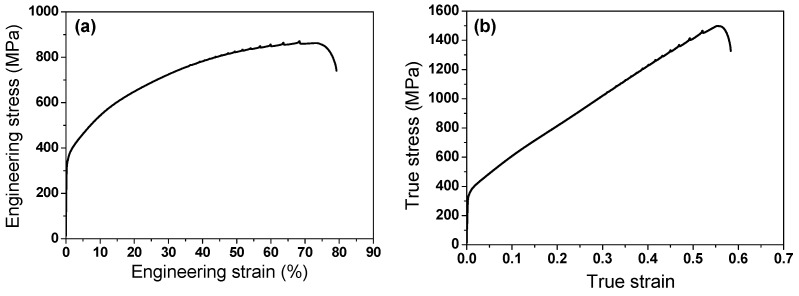
(**a**) Engineering and (**b**) true stress–strain curves of hot-rolled TWIP steel.

Figure 4 presents the tensile curve of the hot-rolled TWIP steel. The steel exhibited an excellent combination of tensile properties: the tensile strength (TS) is 870 MPa and the total elongation (TE) is approximately 80%. However, a small YS compared with TS and a small post-necking elongation compared with TE were observed. These are typical characteristics of TWIP steels [3]. The tensile curve also exhibited a significant serration flow resulting from the DSA [52,53,54].

**Figure 5:**
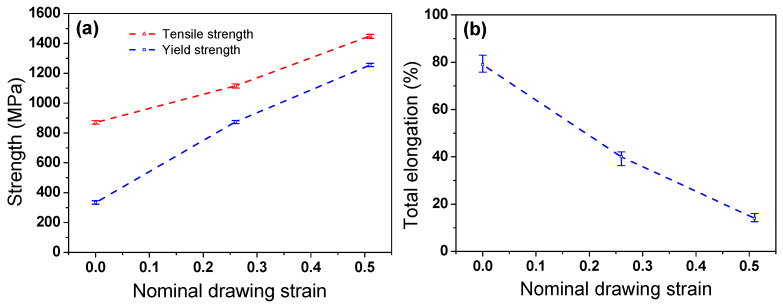
Comparison of (**a**) yield and tensile strengths and (**b**) total elongation of TWIP steel with nominal drawing strain.

Figure 5 compares the YS, TS, and TE of the TWIP steel with *ε*_N_. The YS and TS increased due to the deformation twins and dislocations generated during plastic deformation with *ε*_N_. However, TE decreased sharply with *ε*_N_. The difference between YS and TS was reduced with an increasing *ε*_N_. The specific pattern of increase in strength and the decrease in TE with increasing *ε*_N_ cannot be determined in this study because the tensile test was performed at only three *ε*_N_ of 0.00, 0.26, and 0.51.

### 3.3. Thermophysical Properties

**Figure 6:**
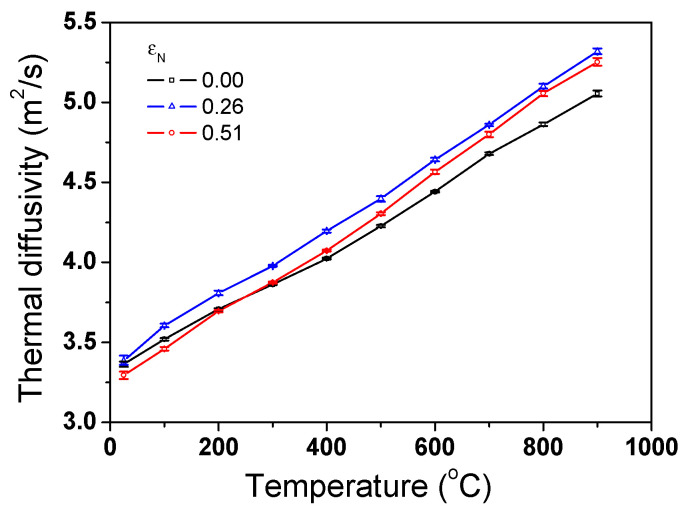
Variations in measured thermal diffusivity of TWIP steel with nominal drawing strain and temperature.

Figure 6 compares the measured *α* of the TWIP steel as functions of temperature and *ε*_N_. Five repeated tests were performed to obtain the one measurement point and error bars were also displayed on the graph. It can be seen that the deviation of *α* values for the repeated measurements is small. It can be clearly observed that α increased with temperature. However, *α* exhibited complex behaviors depending on *ε*_N_. For example, the *α* at an *ε*_N_ of 0.25 exhibited the highest value, regardless of temperature.

**Figure 7:**
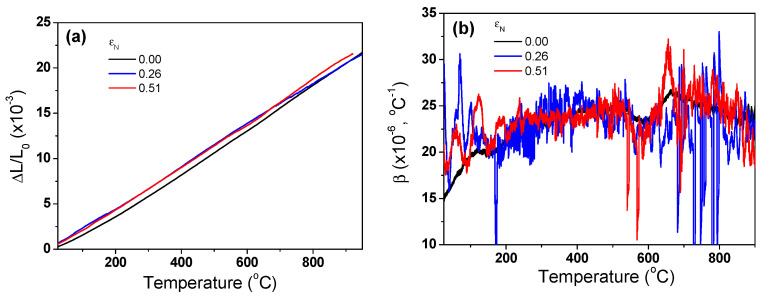
Variations in measured (**a**) thermal expansion ratio of length and (**b**) thermal expansion coefficient of TWIP steel as functions of nominal drawing strain and temperature.

**Figure 8:**
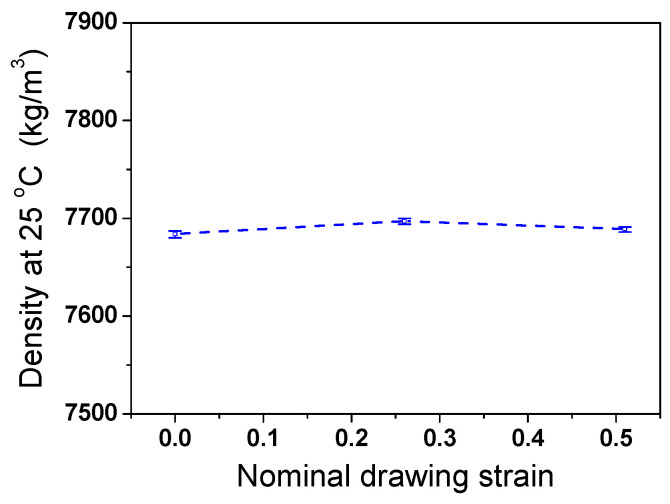
Comparison of measured density of TWIP steel at room temperature with nominal drawing strain.

**Figure 9:**
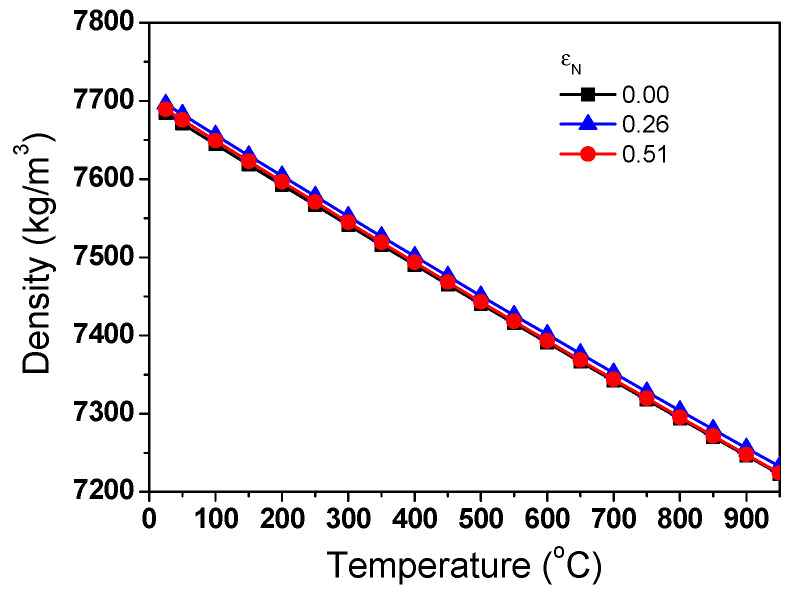
Comparison of calculated density of TWIP steel as functions of nominal drawing strain and temperature.

Figure 7a compares the measured Δ*L*/*L*_0_ of the TWIP steel as functions of temperature and *ε*_N_. It can be seen that the length of the specimen expanded as the temperature increased. All the specimens showed a similar Δ*L*/*L*_0_, regardless of *ε*_N_. The *β* of the TWIP steel was calculated based on Equation (3) as shown in Figure 7b. It was found that *β* exhibited considerable fluctuations with temperature changes due to the significantly small sampling interval. In this measurement, Δ*L* of the specimen was measured every 0.05 °C. This phenomenon appears to be caused by the sensitivity of the probe measuring the Δ*L* in the TMA equipment because this phenomenon was reduced by decreasing the sampling interval and performing a smoothing technique during the data analysis. However, in this study, most of the analyses were conducted focusing on the raw data as shown in Figure 7b to gain more insights on the thermophysical properties. Overall, the *β* of TWIP steel showed no specific patterns with temperature and exhibited similar values, regardless of temperature and *ε*_N_. From another perspective, the overall curves showed a slight increasing trend with temperature. The average *β* of the TWIP steel was approximately 23.5 × 10^−6^ °C^−1^. Based on Refs. [55,56,57,58], the *β* of the TWIP steels was higher than those of plain carbon steels (13–15 × 10^−6^ °C^−1^) and austenitic stainless steels (18–21 × 10^−6^ °C^−1^). Figure 8 shows the measured *ρ*_0_ with *ε*_N_. *ρ*_0_ exhibited similar values, regardless of *ε*_N_. Figure 9 compares the calculated *ρ* using Equation (6). *ρ* decreased with temperature. This result clearly showed that the *ρ* of the TWIP steel was almost independent of *ε*_N_.

**Figure 10:**
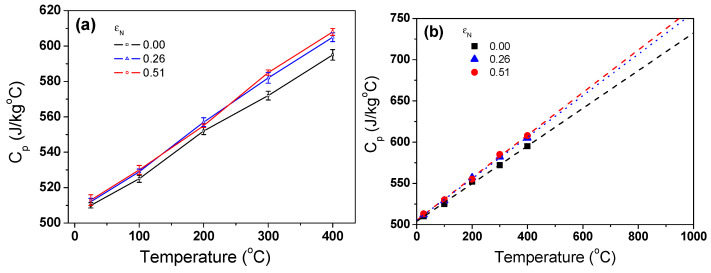
Variations in (**a**) measured and (**b**) linearly fitted specific heat capacity of TWIP steel with nominal drawing strain and temperature.

The *c*_p_ of the TWIP steel increased with temperature as shown in Figure 10a, which is consistent with previous results with stainless steels with no change of crystal structure and magnetic properties [59,60]. Despite some minor fluctuations, the *c*_p_ of the TWIP steel primarily increased with temperature. Therefore, *c*_p_ over the temperature of 400 °C was obtained through linear fitting of the measured values as shown in Figure 10b.

**Figure 11:**
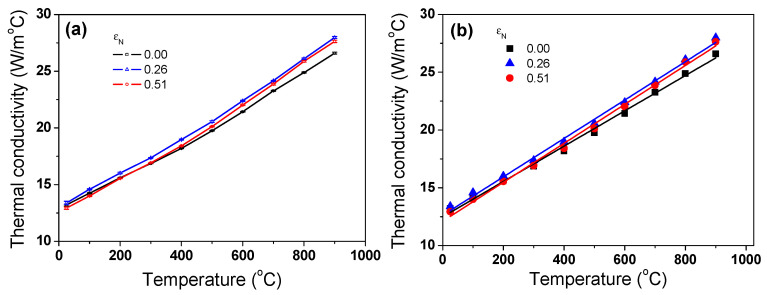
(**a**) Variations in calculated thermal conductivity of TWIP steels with nominal drawing strain and temperature and (**b**) their linear fitted lines.

**Figure 12:**
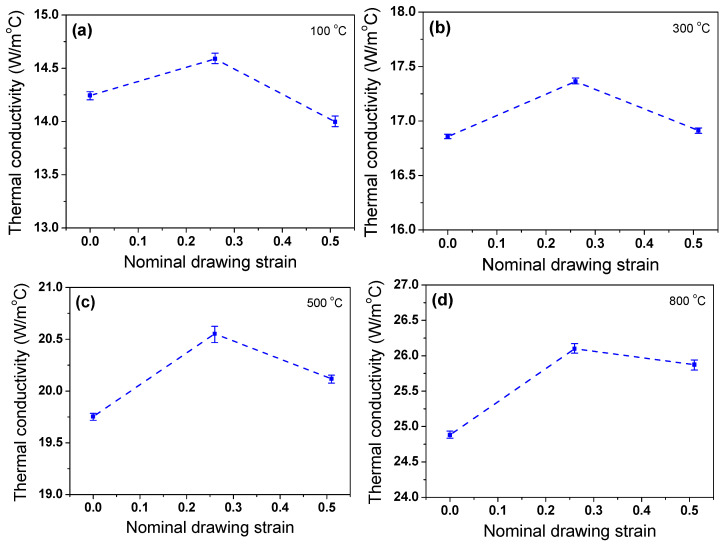
Comparison of thermal conductivity of TWIP steel with nominal drawing strain at temperatures of (**a**) 100 °C, (**b**) 300 °C, (**c**) 500 °C, and (**d**) 800 °C.

The *k* of the specimen with temperature and *ε*_N_ is shown in Figure 11a based on Equation (7). The *k* of the TWIP steel linearly increased with increasing temperature (Figure 11b). This pattern is consistent with the results of stainless steels [10,60], Mg alloys with high impurity [61], and AlCoCrFeNi high entropy alloys [62]. Interestingly, the drawn wire with an *ε*_N_ of 0.26 exhibited the maximum *k* value, regardless of temperature; whereas, hot-rolled wire showed the minimum *k* value, meaning that there is an optimal *ε*_N_ with high *k*. To understand the above phenomenon more effectively, *k* was displayed as a function of *ε*_N_ at a fixed temperature as shown in Figure 12. *k* increased with drawing strain until an *ε*_N_ of 0.26; subsequently, it decreased with *ε*_N_, indicating that *k* exhibited the peak value at an *ε*_N_ of 0.26. Since we only measured *k* value at three *ε*_N_, it is not clear which *ε*_N_ gives the highest *k* value. In other words, the maximum *k* value was known to be around an *ε*_N_ of 0.26, but the exact value is unknown. This is a limitation of this study; therefore, further research is necessary.

## 4. Discussion

It is known that the *k* of metals deteriorates as the amount of plastic deformation increases because the defects, such as dislocations, induced by plastic deformation impede heat flow in the metals [63]. However, it was found that *k* increased in the drawn TWIP steel wire compared with the hot-rolled specimen as shown in Figure 12. And then, *k* decreased again as the amount of deformation increased further. In metals, the energy transport is primarily conducted due to free electrons and lattice waves (phonons) [64]. Therefore, *k* can be expressed as thermal conductivity depending on the movement of free electrons (*k*_e_) and thermal conductivity depending on the influence of phonons (*k*_p_) as follows:

*k* = *k*_e_ + *k*_p_ (9)

**Figure 13:**
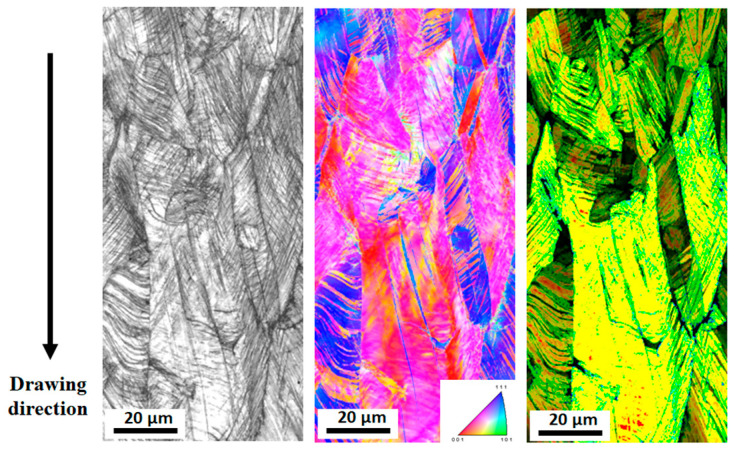
EBSD image quality, inverse pole figure, and grain shape major axis maps of TWIP steel at a nominal drawing strain of 0.51. It was measured by cutting the specimen along the longitudinal direction of drawn wire.

**Figure 14:**
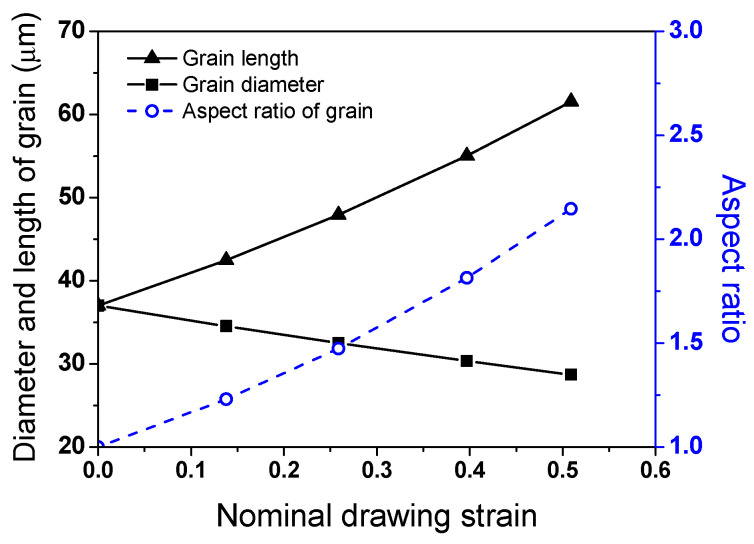
Variations in calculated diameter, length, and aspect ratio of grain in the drawn wire with nominal drawing strain.

As the temperature of metals increases, the greater lattice vibration scatters the free electrons, although the energy of the free electrons and lattice vibrations increase. Under the influence of these two contradictions, the complex behaviors of *k* are observed depending on the temperature of the metals. Dislocations, grain boundaries, solute atoms, and precipitates are the prime scattering centers for free electrons and phonons in metals, which generally lead to the deterioration of *k*. In high-alloyed metals such as stainless steels and TWIP steels, the *k* increased with increasing temperatures, as shown in Figure 11, because the influence of *k*_p_ was stronger compared with *k*_e_ with increasing temperatures. As the amount of strain increases, dislocations accumulate inside the metals, which act as scattering sources for free electrons and phonons, causing *k*_e_ and *k*_p_ to decrease [65]. In this study, the increase in *k* with increasing *ε*_N_ can be explained by the elongation of grain during wire drawing. Figure 13 shows theehEBSD IQ, IPF, and grain shape major axis maps of the present TWIP steel at an *ε*_N_ of 0.51. It was measured by cutting the drawn specimen along the longitudinal direction of wire. It was clearly observed that the grains are elongated along the wire drawing direction. Figure 14 represents the mathematically calculated diameter (*D_grain_*), length (*L_grain_*), and aspect ratio (*AR_grain_*) of grains in drawn wire with *ε*_N_. *ARgrain* is defined as follows:

(10) $${AR}_{grain}=\frac{L_{grain}}{D_{grain}}$$

**Figure 15:**
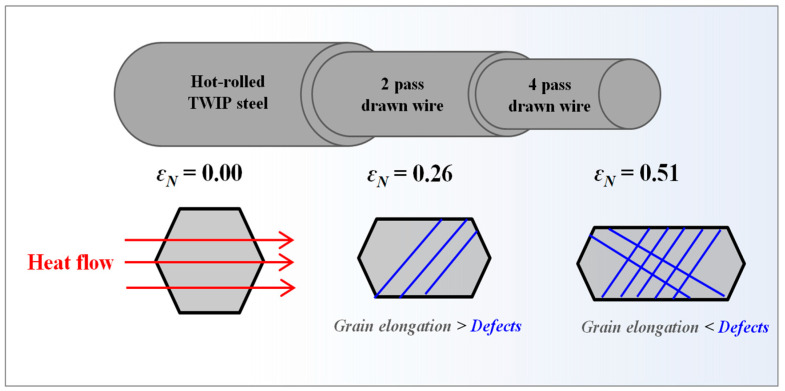
Schematic description of heat flow at grains in drawn wire with nominal drawing strain.

As the wire drawing process progressed, the *L_grain_* increased and the *D_grain_* decreased, leading to an increase in *AR_grain_*. The *AR_grain_* had a value greater than 2.0 at. an *ε*_N_ of 0.51 as shown in Figure 14. This is confirmed by the elongated grains in Figure 13. These elongated grains can increase *k* as the amount of drawing steps increases. Since the grain boundaries along the heat flow increase, the scattering sources of free electrons and phonons increase, leading to the decrease in *k* [66,67]. Meanwhile, Sun et al. [25] and Wang et al. [26] revealed that the electrical conductivity increased in Cu wire and Al-Y alloy by wire drawing, respectively. They explained this mechanism in terms of grain elongation and orientation during wire drawing. Figure 15 shows the schematic description of the heat flow in drawn wire with *ε*_N_. At an *ε*_N_ of 0.26, *k* increased compared with the hot-rolled steel because the increase in *k* due to the grain elongation was greater than the decrease in *k* due to the dislocations generated during wire drawing. However, as the amount of drawing step increased further, the influence of dislocations on *k* increased more than that of the grain elongation, causing *k* to decrease.

Meanwhile, it should be noted that the *k* of TWIP steel was much lower than that of plain carbon steels [11,29,68,69,70,71] owing to the high-alloy contents in the TWIP steel as listed in Table 1. In particular, the difference in *k* between the TWIP and plain carbon steels was extremely large in low temperature ranges. For example, at 100 °C, the *k* of plain carbon steels was approximately 45 W/m°C; in contrast, that of TWIP steel was approximately 14.2 W/m°C as shown in Figure 11. However, all steels showed a similar *k* in high temperature ranges where face centered cubic structure forms. For example, at 1000 °C, the *k* values of TWIP and plain carbon steels showed a similar value, i.e., 28 W/m °C.

**Figure 16:**
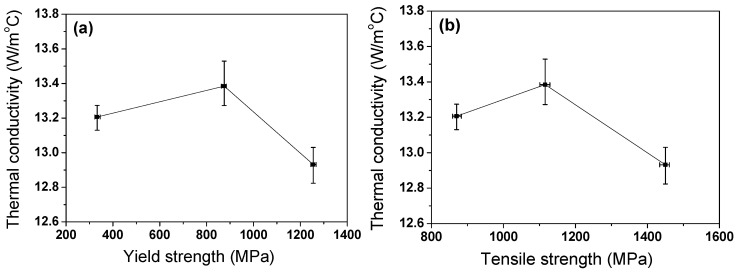
Balance of (**a**) thermal conductivity and yield strength and (**b**) thermal conductivity and tensile strength of drawn TWIP steel wire.

Figure 16 shows the balance of *k* and strength of the TWIP steel with *ε*_N_. It can be seen that there is an appropriate amount of drawing strain that increases both strength and *k*. When the *k* of materials is considered an important factor under service or during the manufacturing process, selecting an appropriate amount of drawing strain can lead to an effective material or process design, which could be of industrial benefit. In addition, based on the present study, care should be taken with the thermal cracks generated in TWIP steels when manufacturing products with TWIP steels using high heat flux processes such as hot working, welding, and heat treatment. The low *k* and high *β* of TWIP steels, compared with plain carbon steels, can induce thermal cracks during high heat flux processes. Accordingly, welding, heat treatment, and hot working processes for TWIP steels must be conducted using different process conditions that should be set appropriately for plain carbon steels.

## 5. Conclusions

Comprehensive study of the relationship between thermal conductivity and the microstructures in TWIP steel wire was performed with strain induced by wire drawing and the results are summarized as follows:

1. The YS and TS increased linearly with increasing drawing strain owing to the deformation twins and dislocations generated during wire drawing. However, TE decreased sharply with drawing strain.
2. The *β* of TWIP steel exhibited a similar value regardless of drawing strain. *ρ* decreased linearly with temperature, and it was almost independent of the drawing strain.
3. *k* initially increased and then decreased after reaching its maximum value with increasing drawing strain. At a nominal drawing strain of 0.26, *k* increased compared with the state of hot rolling because the increase in *k* due to grain elongation was greater than the decrease in *k* due to dislocations during wire drawing. However, as the amount of drawing step increased further, the influence of dislocations on *k* increased more than that of the grain elongation, causing *k* to decrease.

## Figures and Tables

**Table 1:** Main chemical composition and stacking fault energy of test TWIP steel.

Chemical Compositions (wt.%)				Stack Fault Energy in RT (mJ/m^2^)
C	Mn	Al	Fe	
0.70	17.18	1.50	Bal.	31.2

**Table 2:** Process condition of dies for drawing test in the present study.

No. of Pass	Wire Diameter (mm)	Die Angle (°)	R_A_ Per Pass (%)	Total R_A_ (%)	Nominal Drawing Strain
0	15.00		-	-	0.00
1	14.00	12	12.9	12.9	0.14
2	13.18	12	11.4	22.8	0.26
3	12.30	12	12.9	32.8	0.40
4	11.63	12	10.6	39.9	0.51

## Data Availability

The original contributions presented in the study are included in the article, further inquiries can be directed to the corresponding author.
